# Memantine Improves the Disturbed Glutamine and γ-Amino Butyric Acid Homeostasis in the Brain of Rats Subjected to Experimental Autoimmune Encephalomyelitis

**DOI:** 10.3390/ijms241713149

**Published:** 2023-08-24

**Authors:** Beata Dąbrowska-Bouta, Lidia Strużyńska, Marta Sidoryk-Węgrzynowicz, Grzegorz Sulkowski

**Affiliations:** Laboratory of Pathoneurochemistry, Department of Neurochemistry, Mossakowski Medical Research Institute, Polish Academy of Sciences, 5 Pawińskiego Str., 02-106 Warsaw, Poland; bbouta@imdik.pan.pl (B.D.-B.); lidkas@imdik.pan.pl (L.S.); msidoryk@imdik.pan.pl (M.S.-W.)

**Keywords:** EAE, glutamine synthase, PAG, amino acid transport, GABA

## Abstract

Glutamine (Gln), glutamate (Glu), and γ-amino butyric acid (GABA) are essential amino acids for brain metabolism and function. Astrocyte-derived Gln is the precursor for the two most important neurotransmitters in the central nervous system (CNS), which are the excitatory neurotransmitter Glu and the inhibitory neurotransmitter GABA. In addition to their roles in neurotransmission, these amino acids can be used as alternative substrates in brain metabolism that enable metabolic coupling between astrocytes and neurons in the glutamate–glutamine cycle (GGC). The disturbed homeostasis of these amino acids within the tripartite synapse may be involved in the pathogenesis of various neurological diseases. Interactions between astrocytes and neurons in terms of Gln, Glu, and GABA homeostasis were studied in different phases of experimental allergic encephalomyelitis (EAE) in Lewis rats. The results of the study showed a decrease in the transport (uptake and release) of Gln and GABA in both neuronal and astrocyte-derived fractions. These effects were fully or partially reversed when the EAE rats were treated with memantine, a NMDA receptor antagonist. Changes in the expression and activity of selected glutamine/glutamate metabolizing enzymes, such as glutamine synthase (GS) and phosphate-activated glutaminase (PAG), which were affected by memantine, were observed in different phases of EAE. The results suggested perturbed homeostasis of Gln, Glu, and GABA during EAE, which may indicate alterations in neuron–astrocyte coupling and dysfunction of the tripartite synapse. Memantine appears to partially regulate the disturbed relationships between Gln, Glu, and GABA.

## 1. Introduction

Multiple sclerosis (MS) is an autoimmune disease of unknown etiology, in which the immune system attacks the central nervous system (CNS). A characteristic feature of the disease is seen in demyelinating areas in the white matter of the spinal cord and brain that lead to disturbances in neuronal transmission and subsequent neurodegeneration, resulting in the progressive disability of patients [1,2]. Another component of the disease is neuroinflammation. In demyelinating lesions, the presence of lymphocytes, macrophages, and activated microglia was observed in the proximity of the perivascular area, suggesting the involvement of these cell types in the process of demyelination [2,3,4]. The inflammatory process is accompanied by increased levels of inflammatory cytokines and enhanced levels of glutamate released by macrophages and reactive microglia. Enhanced levels of glutamate have been observed in brain lesions and in the cerebrospinal fluid of MS patients [4,5]. Furthermore, correlations between altered glutamate homeostasis, axonal damage, and disturbances in glutamatergic neurotransmission and cell death have been identified during MS pathology and in an established animal model of the disease known as experimental autoimmune encephalomyelitis (EAE) [5,6,7]. The critical involvement of the glutamatergic component in the pathology of MS/EAE has been confirmed by studies indicating the improvement of disease symptoms through the pharmacological inhibition of ionotropic NMDA glutamate receptors with memantine [8,9,10,11].

Increased levels of extracellular glutamate lead to neuronal cell death via excitotoxicity, which is caused by prolonged overactivation of glutamate receptors and the subsequent massive influx of Ca^2+^ into neurons triggering the cascade of pathways leading to cell death [12,13]. Therefore, regulation of the proper glutamate concentration within the synaptic cleft is extremely important.

Glutamate (Glu) along with glutamine (Gln) plays a key role in the brain and is involved in multiple mechanisms. Under normal conditions, the post-synaptic action of Glu is essentially terminated by its rapid uptake by astrocytes that surround the synaptic cleft forming a tripartite synapse. Glu taken up by cells can be then used in general metabolism, protein synthesis, energy metabolism, and ammonia binding or reused as a neurotransmitter. The transfer of Glu from neurons to astrocytes and the subsequent return of Gln from astrocytes to neurons is referred to as the glutamate–glutamine cycle (GGC) [14]. In this cycle, after uptake into astrocytes, Glu is amidated to generate Gln by ATP-dependent glia-specific enzyme glutamine synthase (GS) (EC 6.3.1.2) [15,16,17].

Gln is then released into the extracellular space, taken up by neurons, reconverted to Glu inside glutamatergic neurons [18], and converted to GABA at GABAergic synapses [15]. There is evidence that the pool of Gln for the neuronal synthesis of Glu is of astrocytic origin [19]. The synaptic terminals exhibit abundant activity of phosphate-activated glutaminase (PAG) (L-glutamine aminohydrolase EC 3.5.1.2), which catalyzes the conversion of Gln to Glu. This pathway is present in both glutamatergic and GABAergic neurons [20]. Glutamate decarboxylase (GAD) (L-glutamate 1-carboxylase; EC 4.1.1.15) is involved in the regulation of mammalian brain excitability through synthesis of the major inhibitory neurotransmitter GABA in GABAergic neurons [21].

In addition to their roles in neurotransmission, Gln, Glu, and GABA can be utilized by astrocytes and neurons as alternative metabolic substrates after oxidation to glucose [22,23]. Glu can also be deaminated in astrocytes and converted into α-ketoglutarate, which then enters the tricarboxylic acid cycle [24].

The role of glutamate excitotoxicity in MS/EAE pathology has been previously established and reported [4,25]. Our own studies on the transport of radioactive Glu in fractions of both neuronal (synaptosomes) and glial (GPV fraction) origin isolated from EAE rats under the same conditions as in the present study revealed that the efficiency of Glu uptake into both fractions was significantly enhanced. In addition, the K^+^-stimulated release of accumulated Glu was increased, suggesting an increase in extracellular glutamate levels. The administration of memantine partially ameliorated these changes [10].

The observed disturbances of neuronal and glial transport of Glu in EAE pathology suggest subsequent changes in other amino acids whose metabolism is related to Glu within the tripartite synapse. Although the neuronal and glial transport of glutamate in EAE pathology has been previously examined, the homeostasis of Gln and GABA has been not thoroughly investigated under the conditions of the disease. Therefore, in the present study, we focused on Gln and GABA in the brains of EAE rats to investigate potential disturbances in the transport of these amino acids in brain-derived fractions of both glial and neuronal origin. In addition, the expression and the activity of GS and PAG, which are the main enzymes involved in the metabolism of these amino acids, were studied in different phases of EAE. Since changes in Gln and GABA homeostasis may be secondary to well-known overactivity of glutamatergic neurotransmission during MS/EAE, we also assessed the effect of memantine, an uncompetitive antagonist of ionotropic NMDA glutamate receptors, on the metabolic relationships between Gln, Glu, and GABA in different stages of EAE.

## 2. Results

### 2.1. The Effect of Memantine on the Clinical Course of EAE

The first symptom observed during the onset of EAE was the change in the body weight of the immunized rats compared with the control group. From about day 8 post-immunization (d.p.i.) to 14 d.p.i., EAE rats underwent a progressive weight loss of 20–30% compared with the beginning of the experiment, which corresponds to the acute phase of the disease in which neurological deficits occur. Neurological symptoms of the disease appeared as developmental paralysis of tail and hind limbs and reduced physical activity of experimental rats. The symptoms started to develop at 10–11 d.p.i., peaked at 12–13 d.p.i., and recovered partially at 14 d.p.i. At 17 d.p.i., full recovery of symptoms was observed.

We noted a statistically significant improvement of the condition of the experimental rats after the administration of memantine. This treatment effectively reduced the severity and duration of neurological deficits (Table 1). In the memantine-treated group, all rats attained an improved neurological condition relative to those in the EAE group. The maximal score of neurological deficits decreased to 2.4 ± 0.6 at 12 d.p.i. (flaccid tail, impairment of fighting reflex and/or loss of muscle tone in hind limbs) compared with 4.1 ± 0.2 in untreated rats. In addition, after the administration of memantine, the inductive phase of the disease was extended by a factor of 1.9. Detailed observations of the disease course and the clinical parameters of animals during the experiments, as well as the effects of the memantine administration, are presented in Table 1. Similar effects are also described in detail in our previous publications [8,9,10,11].

### 2.2. Functional Changes in GABA and Gln Amino Acids Transport during the Course of EAE and the Effect of Memantine

The profiles of changes observed in both the uptake and the release of Gln and GABA during the course of EAE were found to be similar in the early phase (4 d.p.i.) and at the peak of the disease (12 d.p.i.). Therefore, we only present graphs corresponding to the acute phase of EAE. In the late stages of the disease, at 20 and 25 d.p.i., the transport of Glu and GABA amino acids returned to control values.

**Figure 1:**
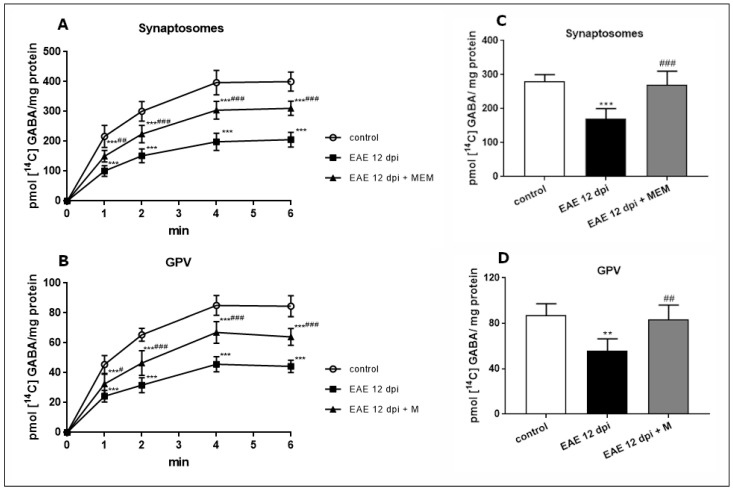
Na^+^-dependent [^14^ C] GABA uptake into synaptosomes (**A**) and GPV fraction (**B**) obtained from control, EAE, and EAE + memantine rat brains during the acute phase of the disease (12 d.p.i.). GABA release from synaptosomes (**C**) and GPV fraction (**D**) obtained from control, EAE, and EAE + memantine rat brain tissue during the acute phase of the disease (12 d.p.i.). Bars represent [^14^C]GABA radioactivity released from pellets after depolarization of membranes with 50 mM KCl at a maximum of the uptake curves (4 min). The radioactivity was assayed 6 min after depolarization. The results represent the means ± SD from six separate experiments performed in triplicate; ** *p* < 0.01, *** *p* < 0.001 significantly different vs. control group; ^#^ *p* < 0.05, ^##^ *p* < 0.01, ^###^ *p* < 0.001 vs. EAE rats not subjected to therapy with memantine (one-way ANOVA with post hoc Tukey’s test).

Uptake of [^14^C]GABA into synaptosomes isolated from EAE rat brains, measured at the maximum of the uptake curve (4 min), decreased by about 50% compared with the controls. Following administration of memantine, the efficiency of uptake improved, although the level of the accumulated neurotransmitter was still lower than in the control rats (by about 25%) (Figure 1A).

With regard to the GPV fraction, the efficiency of GABA uptake was about 70–80% lower (80 pmol GABA/mg protein at 4 min) than in synaptosomes (400 pmol/mg protein at 4 min), indicating the existence of a more active transport system in neurons than in astrocytes. This observation agrees with the results of previous in vitro experiments on cultured neurons and astrocytes (for a review, see [26,27]). However, a similar downward trend was observed. The uptake efficiency was reduced by about 46% in the EAE rats compared with the control rats. The change was partially recovered by administration of memantine (47% vs. EAE) but did not reach the control value (Figure 1B).

In both synaptosomal and glial fractions, K^+^-dependent release of previously accumulated [^14^C]GABA decreased to levels below the control values by 20% and 36%, respectively (Figure 1C,D). After memantine treatment, an improvement of [^14^C]GABA release by about 60% was observed in the synaptosomal fraction relative to the untreated EAE rats (Figure 1C). A similar improvement of about 55% was observed in the GPV fraction (Figure 1D).

**Figure 2:**
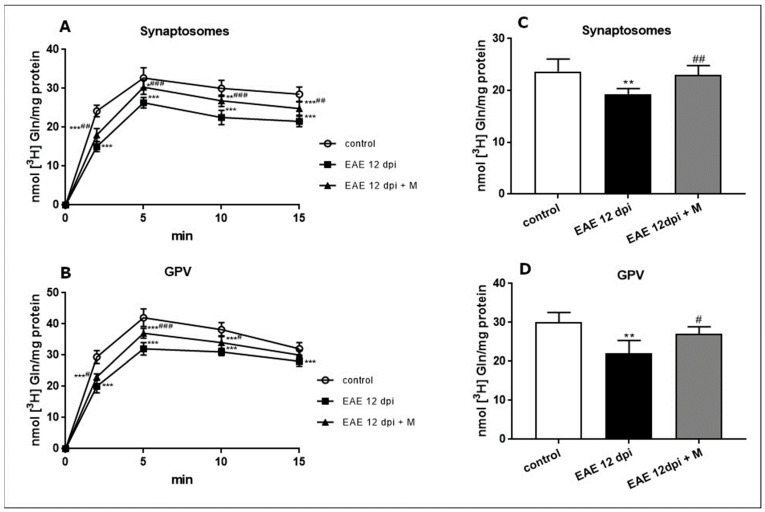
Na^+^-dependent [^3^H]glutamine uptake into synaptosomes (**A**) and GPV fraction (**B**) obtained from the control, EAE, and EAE + memantine rat brain tissue during the acute phase of the disease (12 d.p.i.). Glutamine release from synaptosomes (**C**) and the GPV fraction (**D**) obtained from control, EAE, and EAE + memantine rat brain tissue during the acute phase of the disease (12 d.p.i.). Bars represent [^3^H]glutamine radioactivity released from pellets after the depolarization of membranes with 50 mM KCl at a maximum of the uptake curves (5 min). The radioactivity was assayed 6 min after depolarization. Results represent the means ± SD from six separate experiments performed in triplicate; * *p* < 0.05, ** *p* < 0.01, *** *p* < 0.001 significantly different vs. control group; ^#^ *p* < 0.05, ^##^ *p* < 0.01, ^###^ *p* < 0.001 vs. EAE rats not subjected to therapy with memantine (one-way ANOVA with post hoc Tukey’s test).

Sodium-dependent [^3^H]glutamine transport was measured at the maximum of the uptake curve at 5 min. The uptake efficiency decreased significantly by about 20% in synaptosomal fractions (Figure 2A) and by about 25% in GPV fractions (Figure 2B) of the EAE rats compared with the controls. Treatment with memantine partially reversed these changes in Gln uptake, but the level of accumulated Gln remained lower than in respective controls (Figure 2A,B). The K^+^-stimulated release of Gln decreased significantly in both examined fractions by about 20% and 30% relative to the respective control values (Figure 2C,D). After the administration of memantine, we observed an improvement in the release of previously accumulated [^3^H]glutamine compared with the corresponding controls (EAE rats) (Figure 2C,D).

### 2.3. Changes in Expression of Selected Enzymes Involved in Glutamate–Glutamine Cycle and the Effect of Memantine

Western blot analysis was performed to examine the GS and PAG protein levels in fractions obtained from brain tissue of the control and EAE rats untreated or treated with memantine. Real-time qPCR analysis was used to investigate the changes in the mRNA levels of enzymes.

#### 2.3.1. The Effect of Memantine on the Expression of GS during the Course of EAE

Our previous study indicated an increased number of astrocytes with morphological features of activation in the brain tissue of EAE rats [8]. To determine whether this activation of astrocytes during the course of EAE affects their ability to metabolize glutamate, we assessed the expression of GS (astrocyte-specific marker) in the GPV fraction isolated from rat brains at different phases of EAE and after treatment with memantine.

**Figure 3:**
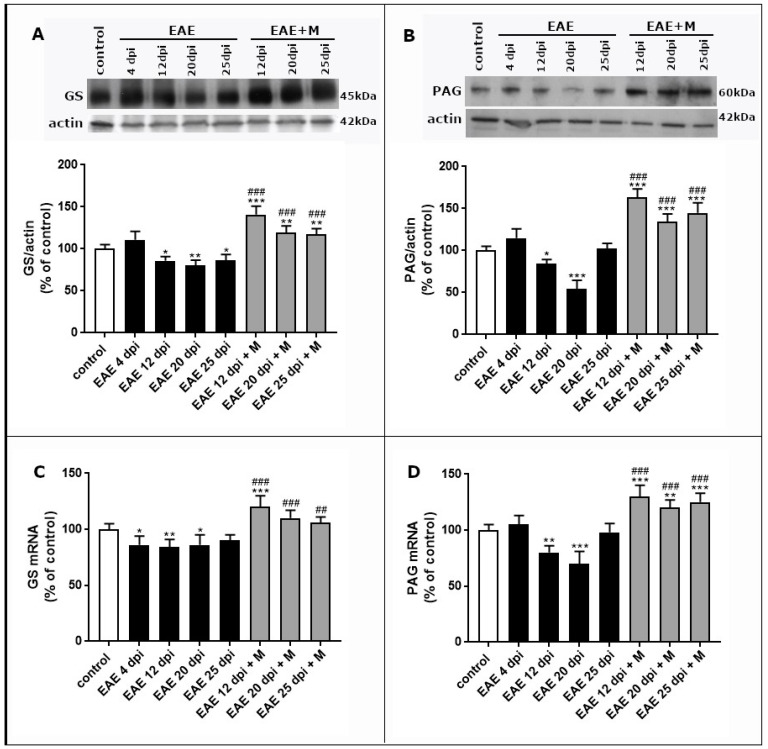
Protein expression of glutamine synthase (GS) (**A**) and phosphate-activated glutaminase (PAG) (**B**) in the brain tissue of control and EAE rats measured at different times post-immunization and after therapeutic treatment with memantine. Representative immunoblots and graphs (**A**,**C**) showing the results of densitometric analysis of four independent immunoblots, normalized to *β*-actin, each performed from a distinct rat brain. Expression of GS and PAG mRNAs (**B**,**D**) in the forebrains of the control, EAE, and EAE rats after treatment with memantine in different phases of EAE. The mRNA levels were determined by quantitative real-time PCR (see Section 4) and normalized to actin. The results are the means from *n* = 6 animals in each group and are expressed as a percentage of the control. * *p* < 0.05, ** *p* < 0.01, *** *p* < 0.001 significantly different vs. control rats; ^##^ *p* < 0.01, ^###^ *p* < 0.001 significantly different vs. EAE rats not subjected to therapy (one-way ANOVA followed by Sidak’s multiple comparison post-test).

The level of GS protein decreased significantly (by about 15–20%) compared with the controls starting from the acute (12 d.p.i.) phase and lasted until the late phase of the disease (25 d.p.i.) (Figure 3A). In all experimental groups after memantine treatment (12–20 d.p.i.), the GS protein level increased by about 60–80% over the control value and by about 30–40% relative to the untreated EAE rats. To further characterize the modulation of GS expression during EAE, the mRNA levels were assessed. The results indicate a statistically significant decrease in GS mRNA in the early (4 d.p.i.), acute (12 d.p.i.), and late (20 d.p.i.) phases of EAE relative to the control group (Figure 3C). After the treatment with memantine, we observed increased levels of GS mRNA in all groups (12–25 d.p.i.) by about 60% compared with the control rats and by 30% compared with the untreated EAE rats at an appropriate time point post-immunization (Figure 3C).

#### 2.3.2. The Effect of Memantine on the Expression of PAG during the Course of EAE

Glutamatergic neurons express high levels of PAG metabolizing enzyme to use glia-derived Gln as a main substrate for Glu synthesis. In the synaptosomal fraction, a strong positive immunoreaction for PAG was observed as a single band near 60 kDa. In the early phase of EAE (at 4 d.p.i.), we did not observe statistically significant changes in the PAG protein levels compared with the control value. At 12 d.p.i. and 20 d.p.i., the protein levels of PAG were decreased significantly by about 20% and 40%, respectively, returning to control levels at 25 d.p.i. (Figure 3B). The administration of memantine increased the expression of PAG protein by about 80% relative to the control values at all time points of the disease (Figure 3B). The profile of changes in PAG mRNA levels corresponded to the observed protein levels (Figure 3D).

### 2.4. The Effect of Memantine on the Activity of Selected Enzymes Involved in Glu Metabolism

To investigate whether the administration of memantine affects the activity of specific enzymes involved in glutamate metabolism (GS and PAG) in different phases of EAE, we used isotopic methods with [^3^H]-labeled substrates (as described in detail in Materials and Methods). The temporal profile of changes in the activity of enzymes was found to be similar in the early (4 d.p.i.) and acute 12 (d.p.i.) phases of EAE. Therefore, we present only one set of results (12 d.p.i.). In the late phase of EAE (20 d.p.i and 25 d.p.i.), the specific activity of both of the investigated enzymes returned to the values observed in the control groups (data are not presented). All rats in the EAE group used for the measurement of the enzyme activity developed maximal neurological deficits (score = 4).

**Figure 4:**
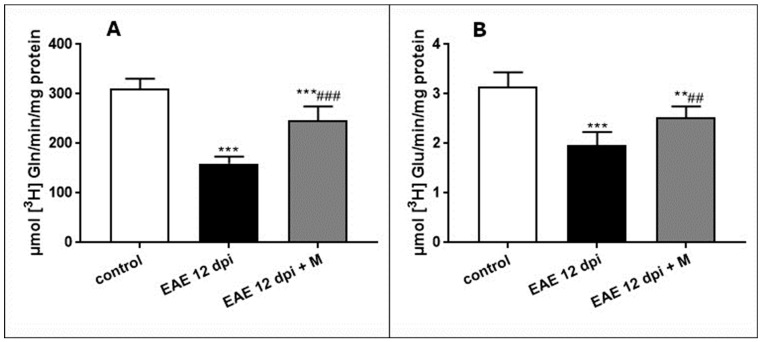
The activity of the astrocyte-specific enzyme glutamine synthase (GS) (**A**) and neuron-specific enzyme phosphate-activated glutaminase (PAG) (**B**) in the brain tissue of control and EAE rats and after therapeutic treatment with memantine at the peak of EAE at 12 d.p.i. All rats in the EAE group without memantine treatment had a neurological symptoms score of 4. The results are the means from *n* = 6 animals in each group and are expressed as means ± SD. ** *p* < 0.01; *** *p* < 0.001 significantly different vs. control rats; ^##^ *p* < 0.01, ^###^ *p* < 0.001 significantly different vs. EAE rats not subjected to therapy (one-way ANOVA followed by Tukey’s multiple comparison post-test).

To test the enzymatic activity of astroglia-specific GS, we compared the ability of the astroglia-derived GPV fraction to convert [^3^H]glutamate into glutamine. In parallel with the overexpression of GS at the protein and mRNA levels observed in subsequent phases of EAE (4 and 12 d.p.i.), the enzyme activity decreased by about 40% below the control values. The administration of memantine partially reversed this effect (Figure 4A).

To investigate neuron-specific PAG activity, the synaptosomal fraction was incubated with [^3^H]-labeled Gln to produced Glu via the PAG reaction. The amount of newly formed Glu was then calculated after elution of the incubation mixture through the Dowex column. The specific activity of [^3^H] glutamate (the product of PAG reaction) in the synaptosomal fraction obtained from EAE rats was markedly lower than that observed in the control synaptosomes (by about 50%). The administration of memantine partially reversed the changes in PAG activity (Figure 4B).

## 3. Discussion

### 3.1. Changes in Metabolism of Amino Acids Involved in Neuron-Astrocyte Crosstalk during EAE

In the present study, we demonstrate changes in the transport of the main amino acids related to Glu metabolism, such as Gln and GABA, in the brain tissue of rats subjected to EAE. We assessed the transport activity of Gln and GABA to indicate the potential availability of these amino acids in both isolated nerve endings and the glial fraction under the conditions of excitotoxicity observed during the course of EAE. In addition, the expression and activity of the selected enzymes (GS, PAG) involved in brain Glu/Gln metabolism were assessed. This work complements our previous studies [8,9,10,11] by using a comparable approach to study the effects of memantine on Gln and GABA metabolism.

The use of cell fractions of neuronal origin (synaptosomes) and glial origin (glial plasmalemmal vesicles GPV) in biochemical measurements enables the modeling of neuron–astroglia interactions in ex vivo experiments. Such an approach may be complementary to in vitro studies using cell co-cultures. Synaptosomes obtained using the procedure described by Booth and Clark [28] are characterized by high purity and well-maintained energy metabolism and are, therefore, considered a good model of nerve endings involved in transport assays. The GPV fraction obtained using the method of Daniels and Vickroy [29] was validated by the authors for structural integrity and analyzed for glial markers and functional specificity, providing solid evidence of the glial nature. The activity of the marker enzyme glutamine synthetase (GS) was also demonstrated in the GPV fraction [30]. Our own morphological examination revealed a large number of membrane-encapsulated vesicles that can be classified as small spherical structures and large irregularly shaped structures [31] expressing the astrocyte-specific marker GFAP [32]. This fraction was used to analyze amino acid transport and GS activity.

The contribution of the glutamatergic component to MS pathology has been previously confirmed by the observation of increased levels of glutamate in the cerebrospinal fluid of patients with progressive neurological disabilities or the presence of excitotoxicity-induced neurodegeneration in an animal model of the disease [5,6,7].

The results of the current study indicated impairment of Gln transport in the early and acute (4–12 d.p.i.) phases of EAE in both synaptosomes and GPV fractions. Furthermore, a kinetic study of GABA transport showed that both the neuronal GABA uptake and release were significantly lower during the disease. The observed changes are consistent with previously reported impairment in Glu homeostasis within the tripartite synapse [10] and may be secondary to the dysfunction of astrocytes. This pool of cells is known to undergo early (4 d.p.i.) [32] and late (12 d.p.i.) [8] activation in the brain of EAE rats, suggesting a significant contribution of these cells to EAE pathology. In this context, reactive astrocytes may become dysfunctional, thereby affecting Glu, Gln, and GABA metabolism within the tripartite synapse. The observed lower activity of the astrocytic enzyme GS, which is crucial for amino acids homeostasis, is proof of this concept.

In normal brain astrocytes, GS catalyzes the amidation of Glu to Gln. Through this conversion, GS helps to maintain the proper balance of Glu and ammonia, controlling the excitotoxicity caused by the increased glutamate to glutamine ratio. The reduced activity of GS in different phases of EAE indicates a reduced ability of the enzyme to convert Glu into Gln and, consequently, an increase in the level of extracellular Glu, leading to the excitotoxicity phenomenon. Our observation is consistent with previous results showing reduced GS activity in mouse models of EAE related to the severity of the disease [33,34]. Moreover, in the course of the disease, we observed a decrease in both GS mRNA and protein, which may be related to reduced enzymatic activity.

PAG is another enzyme which is critically involved along with Gln in the Glu synthesis pathway in neurons, although not specifically in glutamatergic neurons. PAG is located in the mitochondrial compartment and is strongly inhibited by the reaction product, Glu [35,36]. Therefore, the reduced activity of PAG observed by us during EAE may be the result of feedback regulation caused by elevated levels of Glu. Another possibility is that the loss of GS and PAG enzymatic functions may be related to oxidative stress, which is known to play a role in many aspects of MS/EAE pathogenesis, including demyelination, axonal injury, and loss of integrity of the blood–brain barrier (BBB). Oxidative stress and ROS/NOS production have been reported to increase during EAE [11,37,38], leading to the oxidation of proteins, including enzymes. The oxidation of GS has been shown to be associated with the decreased enzyme activity and increased glutamate/glutamine levels [33].

Regardless of the cause, decreased PAG activity, along with decreased ability to transport Gln into nerve endings, has the potential to reduce levels of extracellular Glu by limiting the availability of the substrate and efficiency of the synthesis reaction. In parallel with the decreased activity, PAG was observed to be persistently downregulated during the course of EAE, except in the early phase (4 d.p.i.) of the disease.

It is also worth noting that the limited availability of Gln and PAG also means that limited amounts of GABA are formed in GABAergic synapses and finally taken up by astrocytes. We observed a decreased efficiency of GABA transport in both the synaptosomal and glial fractions isolated from EAE brain tissue. This suggests downregulation of GABA-mediated signaling from neurons to astrocytes followed by an imbalance between inhibitory and excitatory neurotransmission that accelerates existing overexcitation.

It should be also mentioned that during the astro-synaptic turnover of Glu and Gln, certain amounts of these amino acids taken up by astrocytes are involved in oxidative and glycogenolytic metabolism, thus supporting key aspects of astrocytic energetics [39]. Importantly, the metabolic fate of Glu in astrocytes is concentration dependent, which means that with increasing concentrations of extrasynaptic Glu, the fraction metabolized increases, while that converted to Gln decreases [40].

### 3.2. The Protective Effect of Memantine on Disturbed Homeostasis of Amino Acids Involved in Neuron–Astrocyte Crosstalk

The potential use of glutamate NMDA receptor antagonists such as memantine as neuroprotective agents has been established in both preclinical and clinical studies [41]. Unlike other NMDAR channel blockers, memantine is well tolerated by patients and does not exhibit serious side effects [42]. This weak uncompetitive antagonist has been approved by the European Union and the U.S. Food and Drug Administration (FDA) for the treatment of dementia and Alzheimer’s disease.

As previously reported, it can also be used as a pharmacological agent to restore the physiological function of synaptic neurotransmission and to produce a neuroprotective effect against excitotoxicity-induced neurodegeneration during EAE. It also has a positive effect on the inflammatory process and oxidative stress during EAE [8,9,10,11]. By affecting the expression and function of NMDARs, memantine significantly improves the general condition of EAE rats [9] and positively affects the expression and function of glutamate transporters (GluTs) and the activity of Glu transport in neuronal (synaptosomes) and glial (GPV) fractions during the acute phase of EAE [10], thus proving the participation of the glutamatergic component in the pathology of the disease.

The current results indicate that it also positively regulates disturbed neuron–astrocyte interactions in terms of Glu, Gln, and GABA transport and metabolism, to some extent. By blocking NMDA receptors, memantine accelerates Glu metabolism by increasing astrocytic expression and activity of GS. The improvement of enzyme activity increases the efficiency of Gln synthesis and the feedback effect of overactivation of Gln transport in fractions of both glial and neuronal origin. The greater availability of Gln in nerve endings, together with the increased expression and function of its PAG metabolizing enzyme, provides an efficient source of both Glu and subsequently GABA, which is transported more effectively into synapses as well as astrocytes.

In conclusion, this work demonstrates changes in neuron–astrocyte crosstalk during EAE as reflected by the disturbed relationships between Glu, Gln, and GABA, starting from the early phase and continuing in the subsequent phases of the disease. We observe dysfunction of Gln and GABA transport and decreased expression and activity of the Glu and Gln metabolizing enzymes glutamine synthase (GS) and phosphate-activated glutaminase (PAG), respectively. The reduced activity of GS suggests that excitotoxicity may occur in the brain of EAE rats following the reduction of Glu removal by astrocytes. In turn, decreased GABA transport suggests impairment of GABA-mediated neuron–astrocyte signaling.

The administration of memantine to EAE rats contributes to the overexpression and overactivity of examined enzymes and increased efficiency of amino acid transport in fractions of both neuronal and glial origin. By the partial reversal of the neurological deficits and observed biochemical changes, memantine significantly improves the general condition of the animals.

Thus, our results confirm the implication of the glutamatergic component in pathological mechanisms operating during EAE by demonstrating that the suppression of glutamate excitotoxicity with memantine regulates amino acid homeostasis in the tripartite synapse and provides a partial neuroprotective effect.

Whether this neuroprotective potential can be translated into clinical practice has not yet been fully confirmed. Current clinical trials on the potential use of memantine in the treatment of MS are insufficient. The use of this drug as a therapeutic tool still requires thorough studies in terms of the different MS subtypes, the effects of concomitant use of other therapies, and the duration of the memantine administration [43].

## 4. Materials and Methods

### 4.1. Animal Model

Eight-week-old, female Lewis rats (183 ± 10 g) were supplied by the animal house of the Mossakowski Medical Research Centre, Polish Academy of Sciences. All procedures involving rodents were carried out in accordance with the European Communities Council Directive (86/609/EEC) for the Care and Use of Laboratory Animals and in accordance with ethical guidelines for the care and use of laboratory animals. These procedures were approved by the IV Local Care of Experimental Animal Committee in Warsaw (61/2009). The rats were housed in a temperature-controlled room with a 12 h light/dark cycle and free access to water and food, including the Ssniff^®^ R-2 complete diet for rat breeding (Ssniff Spezialdoten GmbH, Soest, Germany).

All procedures using animals were performed under sodium pentobarbital anesthesia to minimize suffering. To induce EAE, the rats were immunized subcutaneously in both hind feet with 100 µL of inoculum containing guinea pig spinal cord homogenate emulsified in Freund’s complete adjuvant (CFA) containing 5.5 mg/mL *Mycobacterium tuberculosis* H37Ra (Difco, Detroit, MI, USA). The control group received inoculum containing CFA without spinal cord homogenate [44,45]. The animals were observed daily and monitored for weight and neurological deficits, and the clinical severity was scored. The clinical scores of EAE were assigned according to the following criteria: 0, asymptomatic; 1, complete loss of tail tone; 2, hind limb paraplegia; 3, complete hind limb paralysis; 4, hind limb paralysis with forelimb involvement; and 5, moribund/dead [44,45,46].

### 4.2. Experimental Groups and Tissue Processing

The rats were arranged into eight groups (one control and seven experimental groups sacrificed at various phases of EAE and with different recovery periods after treatment with memantine): group I, control (healthy); group II, EAE 4 d.p.i.; group III, EAE 12 d.p.i.; group IV, EAE 20 d.p.i.; group V, EAE 25 d.p.i.; group VI, EAE 12 d.p.i. + memantine; group VII, EAE 20 d.p.i. + memantine; and group VIII, EAE 25 d.p.i. + memantine. Each group consisted of 15 animals. During the experiments, the rats were monitored until days 4, 12, 20, and 25 after the initial injection of the EAE-inducing inoculum. At the respective time points, six rats from each group were sacrificed for the preparation of synaptosomal (*n* = 6) and GPV (*n* = 6) fractions. Both fractions were prepared from fresh brain tissue homogenates, stored on ice and used on the same day for the measurements of neurotransmitter transport (uptake and release). The other brains were quickly removed, frozen in liquid nitrogen, and stored at −80 °C for further experiments that included extraction of RNA or preparation of tissue homogenates. To obtain homogenates for immunoblots, forebrain tissue was homogenized in 50 mM phosphate buffer (pH 7.4) containing 10 mm EGTA, 10 mM EDTA, 0.1 mM PMSF, and 10 mM NaCl in the presence of the protease inhibitor cocktail (1 µg/mL leupeptin, 0.1 µg/mL pepstatin, and 1 µg/mL aprotinin).

The glutamate receptor antagonist memantine was administered at a dose of 60 mg/kg b.w./day. Memantine was dissolved in PBS and administered intraperitoneally to the EAE rats once daily for 7 consecutive days, starting from day 5 post-immunization (p.i.) until day 11 p.i. as described previously [11].

### 4.3. Preparation of Synaptosomes

The synaptosomal fractions were isolated from the forebrain of rats using a discontinuous Ficoll gradient (7%, 12%) at 99,000× *g* as described by Booth and Clark [28]. The final synaptosomal pellet was washed once in Krebs–Ringer buffer (140 mM NaCl, 5 mM KCl, 10 mM Tris-HCl, 1.4 mM MgSO_4_, and 1 mM Na_2_HPO_4_; pH 7.4), centrifuged, and suspended in Krebs–Ringer buffer to obtain a protein concentration of approximately 1 mg/mL. The synaptosomes obtained by this procedure were highly pure and maintained their energy metabolism. Therefore, they were considered to be a good model of nerve endings [28]. Fractions were used for the [^3^H] glutamine and [^14^C] GABA transport (uptake and release) measurements.

### 4.4. Preparation of GPV Fraction

The glial plasmalemmal vesicles (GPV) fractions were isolated according to the method of Daniels and Vickroy [29] with slight modification as described in our previous papers [10,31,47]. Briefly, the brain tissue was homogenized in 30 mL of isolation medium (0.32 M sucrose and 1 mM EDTA) and centrifuged at 1000× *g* for 10 min. The supernatant was diluted using SEDH medium (0.32 M sucrose, 1 mM EDTA, 0.25 mM dithiothreitol, and 20 mM HEPES, pH 7.4) and centrifuged at 5000× *g* for 15 min. After several additional fractionations, the material was centrifuged in a three-step discontinuous Percoll gradient (20%: 10%: 6%) for 10 min at 33,500× *g*. The layer between 0% and 6% Percoll was collected to obtain GPV used for further examination of [^3^H] glutamine and [^14^C]GABA transport.

### 4.5. Transport of Amino Acid Neurotransmitters in Brain Synaptosomes and GPV Fractions

The protein concentration in both fractions was determined by the method of Lowry [48]. Synaptosomal and GPV fractions were used to measure Na^+^-dependent [^3^H] glutamine and [^14^C]GABA uptake and KCl-dependent release of accumulated amino acids. The measurement of radioactive glutamine accumulation was performed according to the filtration method described by Divac [49]. Radioactivity trapped on the filters was then measured in a liquid scintillation counter (Wallack, 1409). In case of the release, 50 mM KCl was used at a maximum of the uptake curves (6 min), and liberated radioactivity was assayed after 6 min. To prevent the conversion of glutamate to α-ketoglutarate, aminooxyacetic acid (AOAA), which is an inhibitor of AAT (aspartate aminotransferase), was added.

Synaptosomal and GPV fractions were used for radioactive [^14^C]GABA uptake and release. The uptake of GABA was strongly Na^+^ dependent [50]; therefore, the measurements were performed only in sodium containing buffer according to the method described by Troeger et al. [51]. Synaptosomes and the GPV fraction were suspended at about 1 mg protein/mL and were preincubated in Krebs–Ringer buffer, pH 7.4, with 2.5 mM CaCl_2_ and 10 mM glucose at room temperature for 5 min. The incubation mixture contained 0.2 mM AOAA in order to inhibit the enzymatic degradation of GABA. The reaction was started by adding [^14^C] GABA (f.c. 2 µM; specific activity 22.6 mCi/mmol). Samples of the incubation mixture were withdrawn at the desired time intervals and centrifuged through silicon oil (specific gravity 1.03, General Electronic, Boston, MA, USA) on a Beckman microfuge. Pellets were solubilized with NCS tissue solubilizer, and the radioactivity was counted using Bray scintillation fluid. For the release studies, 50 mM KCl was added to the incubated fraction (synaptosomes or GPV) at 6 min, at the maximum of the uptake curve, and the samples were centrifuged as above after 6 min.

Glutamine transport to the synaptosomes and GPV fraction was assayed in the incubation mixture containing 140 mm NaCl, 5 mM KCl, 1 mM MgCl_2_, 1.2 mM NaHPO_4_, 10 mM glucose, and 20 mM HEPES, pH 7.4, and 50 µL of subcellular fraction. After incubating the samples at 37 °C for 5 min, uptake was initiated by adding 50 µM of radioactive glutamine (31.9 mCi/mmol [^3^H]glutamine). At various time intervals, the samples were filtered through the glass fiber filters, using a vacuum filtration system. The filters were washed with two portions of ice-cold buffer and placed in Triton X-100 for 20 min. The radioactivity trapped on the filters was counted in scintillation Bray fluid. Only the total uptake of glutamine was measured, without distinguishing the type of Gln entry. For the release studies, 50 mM KCl was added to the incubated fraction as described above.

### 4.6. Western Blot Analysis

Sodium dodecyl sulfate-polyacrylamide gel electrophoresis was performed according to Laemmli [52]. Samples containing 30 µg protein were prepared in loading buffer (Laemmli buffer 1:1), separated on 10% polyacrylamide gel, and transferred to nitrocellulose membrane (Hybond-C extra, Amersham, Germany). Proteins were fixed on the membrane in TBS buffer containing 0.1% Tween-20 and 5% non-fat milk (TTBS) for 1.5 h. After washing in TTBS buffer (3 × 10 min), the blots were incubated first with primary monoclonal antibodies against PAG (rabbit, Invitrogen, 1:2000, Waltham, MA, USA) and GS (rabbit, Sigma, 1: 20,000, St. Louis, MO, USA) for 2 h and then with secondary antibodies conjugated to HRP (1:10,000, Sigma). Bands were visualized using the chemiluminescence ECL kit (Amersham) and exposed for 1–5 min to Hyperfilm ECL (Amersham). The PAG and GS immunocontent was determined by densitometric analysis using UltroScan^+^ XL (Pharmacia, Uppsala, Sweden).

### 4.7. qPCR Analysis

The total RNA was extracted from the brain cortex of all experimental groups of animals. Isolation was performed using TRI Reagent (Sigma, St. Luis, MO, USA) according to the method of Chomczynski [53]. RNA (2 µg) was reverse transcribed using random primers and AMV reverse transcriptase (Life Technologies, Forest City, CA, USA). The RT-PCR conditions induced reverse transcription at 42 °C for 45 min followed by denaturation at 94 °C for 30 s. TaqMan assays were used for quantitative real-time PCR analysis. Rat specific primers for GS *Rn01437204_m1* and PAG *Rn00710600_m1*, from Life Technologies, Forest City, CA, USA, were used. The levels of enzymes and actin mRNA were determined using TaqMan assay reagents (Life Technologies, Forest City, CA, USA). Quantitative real-time PCR (qPCR) analysis was conducted on a Roche LightCycler^®^ 96 system using 5 µL of RT product, TaqMan PCR Master Mix primers, and a TaqMan probe at a total volume of 20 µL. The cycle conditions for the qPCR were as follows: initial denaturation at 95 °C for 10 min, 45 cycles of 95 °C for 15 s, and 60 °C for 1 min. Each sample was analyzed in triplicate. The relative expression of the enzyme mRNA was normalized to actin (Actb) as a reference gene and calculated on the basis of the _∆∆_Ct method.

### 4.8. Measurement of GS Activity

The activity of the enzyme in glial GPV samples was determined according to the method of Pishak and Philips [54]. The reaction mixture contained equal amounts (5 µL) of 500 mM imidazole-HCl, 150 mM MgCl_2_, 100 mM ATP, 40 mM NH_4_Cl, 10 mM dithioerythritol (DTE), and L-[G-^3^H] glutamic acid (specific activity 1.06 mCi/mmol) mixed with unlabeled glutamic acid to a concentration of 75 mM. After the addition of 20 µL of the GPV fraction, the reaction mixture was incubated for 30 min at 37 °C. The reaction was stopped with 1 mL of cold water and transferred to two stacked ion exchange columns (Dowex-l-H^+^ on top of Amberlite CG-50, H^+^) to separate the reaction product ([^3^H]glutamine) from the other labeled compounds. Each column was eluted with 3 mL of cold water. The eluent was collected, and the radioactivity was measured. The results were expressed as a specific activity (nmole [^3^H]glutamine per min per mg protein).

### 4.9. Measurement of PAG Activity

The activity of the enzyme was measured in synaptosomes, according to the method of Shapiro et al. [55]. The assay was performed at 37 °C with 60 µL of a solution containing 0.1 µM of radioactive glutamine: L-[3,4-^3^H(N)], specific activity 31.9 Ci/mmol in a buffer containing 150 mM potassium phosphate, 0.2 mM EDTA, 20 mM Tris-HCl, and 20 mM glutamine, pH 8.6. The reaction was initiated by the addition of 10 µL of synaptosomes (subjected earlier to fractionation with Triton X-100). After 10 min of incubation, the samples were mixed with 1 mL of 20 mM imidazole buffer, pH 7.0, containing 30 mM glutamine and 3 mM glutamate and applied to the columns with Dowex AG 1-X2 for the separation. The resting glutamine radioactivity was then washed out with four portions of imidazole buffer containing 30 mM glutamine. The PAG reaction product, [^3^H]glutamate, was eluted with 2 mL of 30 mM HCl and collected in scintillation vials with Bray scintillation fluid for counting. The results were expressed as specific activity (µmoles glutamate per min per mg protein).

### 4.10. Statistical Analysis

The results were expressed as means ± SD from three to seven experiments as stated in the figure legends. The statistical significance was assessed by one-way ANOVA with Tukey’s or Sidak’s multiple comparison tests to identify the changes that were significantly different from the values of the control or untreated EAE rats using GraphPad PRISM software, version 6.0 (San Diego, CA, USA).

## Figures and Tables

**Table 1:** Characteristic of the EAE model and clinical parameters of EAE rats prior to and after treatment with memantine at different stages of the disease.

	Control	EAE				EAE + Memantine		
		4 d.p.i.	12 d.p.i.	20 d.p.i.	25 d.p.i.	12 d.p.i.	20 d.p.i.	25 d.p.i.
Animals with severe EAE (%)	-	0	100	0	0	73	0	0
Inductive phase (days)	-	-	9 ± 2	-	-	11 ± 1 ^#^	-	-
Average score	-	0.2 ± 0.3	4.1 ± 0.2	2.2 ± 0.6	0.4 ± 0.5	2.4 ± 0.6 ^##^	1.2 ± 0.6 ^##^	0.2 ± 0.3
Cumulative CI (score)	-	2.5	61.5	33.5	6.5	36.0 ^##^	17.5 ^##^	2.5 ^##^
Number of animals	15	15	15	15	15	15	15	15

Administration of NMDAR antagonist memantine reduced the neurological deficits and improved the condition of the experimental rats during the course of EAE. The values represent the means ± SD from *n* = 15 animals in each experimental groups, ^#^ *p* < 0.05, ^##^ *p* < 0.01 significantly different vs. EAE untreated rats (one-way ANOVA with post hoc Sidak’s test).

## Data Availability

The data presented in this study are available at the Laboratory of Pathoneurochemistry, Department of Neurochemistry, Mossakowski Medical Research Institute, Polish Academy of Sciences.
